# Supplementary material for: Investigating the Impact of Sample Preparation on Mass Spectrometry-Based Drug-To-Antibody Ratio Determination for Cysteine- and Lysine-Linked Antibody–Drug Conjugates
Source: Antibodies (Basel). 2020 Sep 8;9(3):46. doi: 10.3390/antib9030046 (PMC7551423; doi:10.3390/antib9030046)
Supplement: Supplementary file 1 [file antibodies-09-00046-s001.zip › antibodies-833409-supplementary.docx]

# Supplementary material

Investigating the Impact of Sample Preparation on Mass Spectrometry-Based Drug-To-Antibody Ratio Determination for Cysteine- and Lysine-Linked Antibody–Drug Conjugates

Malin Källsten ^1,2,^*, Rafael Hartmann ^3^, Lucia Kovac ^2^, Fredrik Lehmann ^4^, Sara Bergström Lind ^1^ and Jonas Bergquist ^1,^*

^1^ Department of Chemistry-BMC, Uppsala University, S-75124 Uppsala, Sweden; [mailn.kallsten@kemi.uu.se](mailto:mailn.kallsten@kemi.uu.se), [sara.lind@kemi.uu.se](mailto:sara.lind@kemi.uu.se), jonas.bergquist@kemi.uu.se

^2^ Recipharm OT Chemistry AB, Uppsala, Sweden; lucia.kovac@recipharm.com

^3^ Department of Medicinal Chemistry, Uppsala University, Uppsala; Sweden; rafael.hartmann@ilk.uu.se

^4^ Oncopeptides AB, Stockholm, Sweden; fredrik.lehmann@otpharma.se

***** Correspondence: malin.kallsten@kemi.uu.se (M.K.); jonas.bergquist@kemi.uu.se (J.B.); Tel.: +46-(0)18-4713696 (M.K.); +46-(0)18-4713675 (J.B.)

## Interchain disulfide reduction


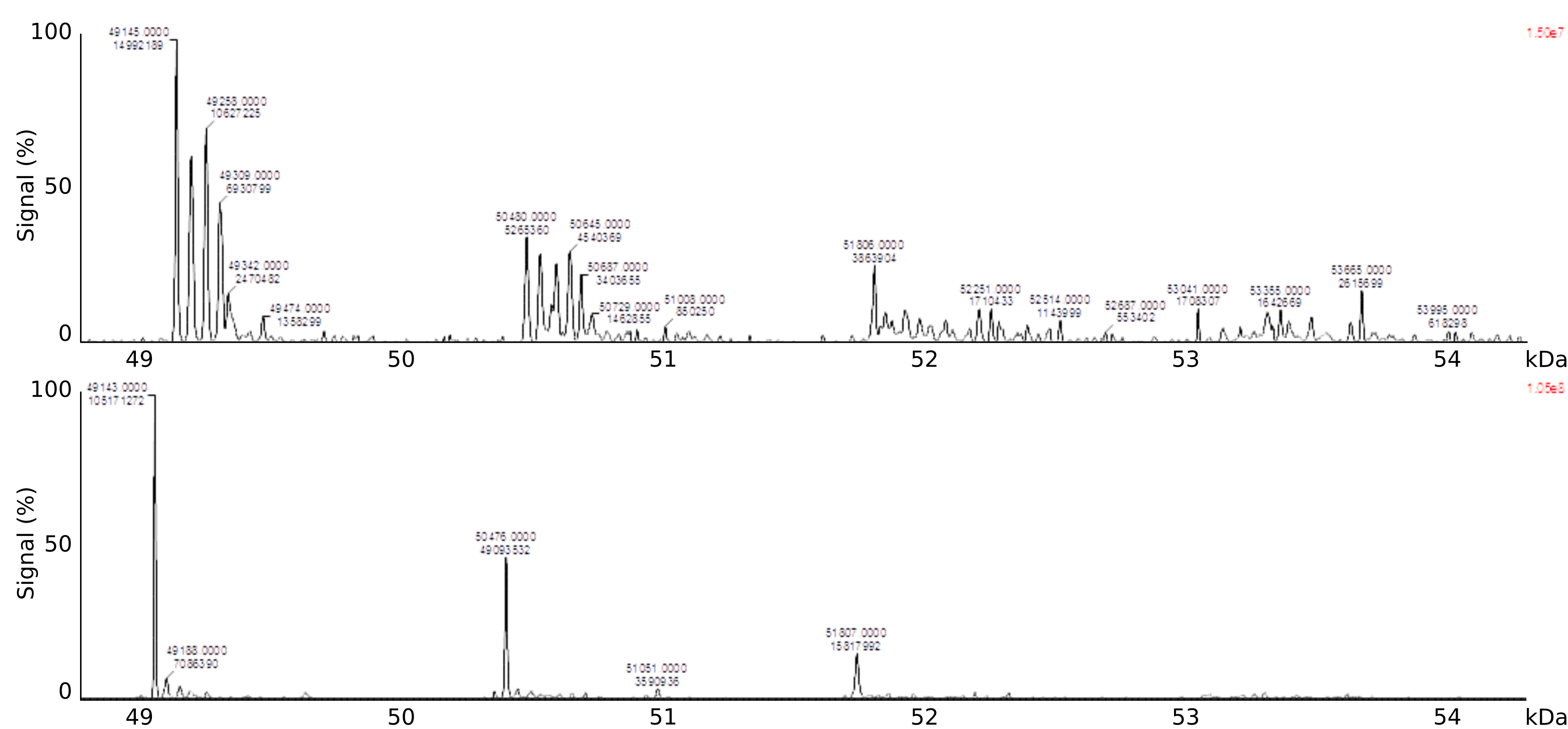


Figure S - 1. Deconvoluted heavy chain from vcMMAE-Trastuzumab before the addition of alkylation agent iodoacetamide (bottom) and after adding alkylation agent (top) after interchain disulfide reduction by dithiothreitol to prevent reformation of the disulfide bridges.


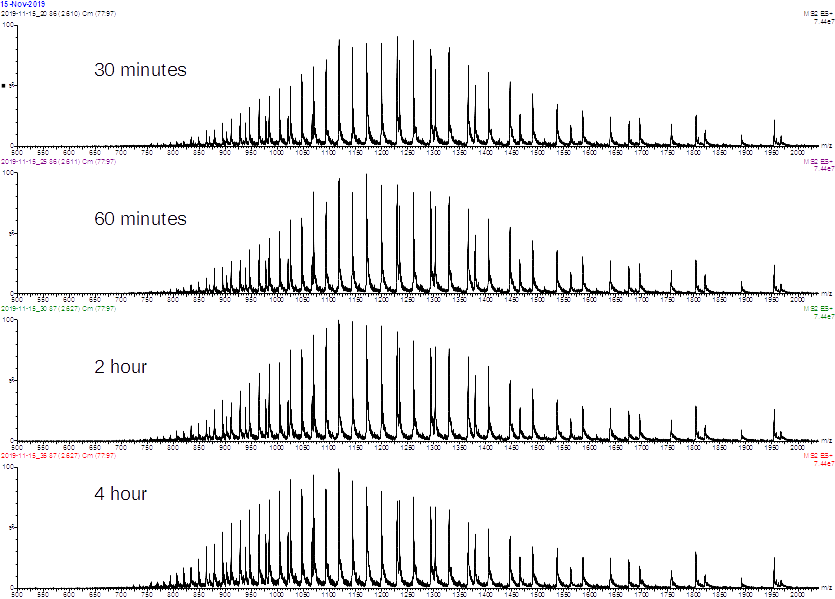


Figure S - 2. Detected charge envelope and Trastuzumab for samples subjected to reduction for 30 minutes, 60 minutes 2 hours and 4 hours.


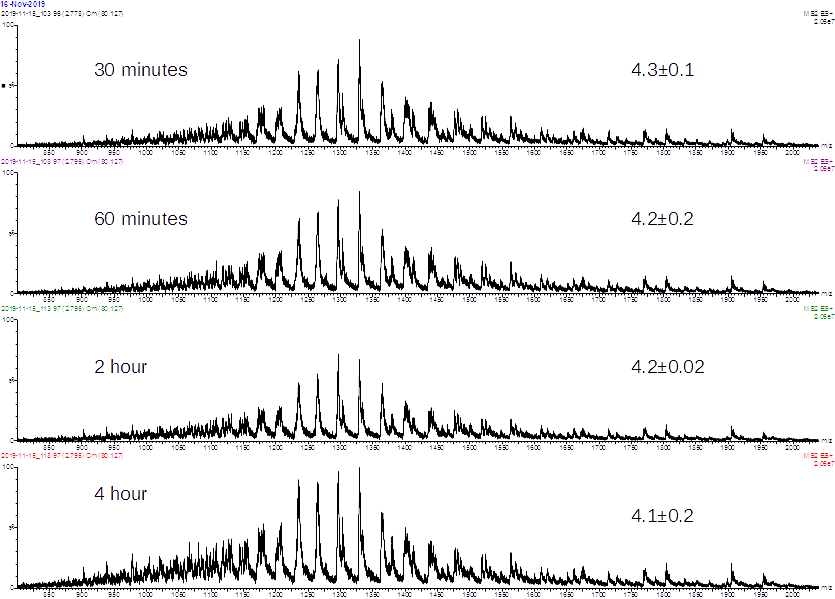


Figure S - 3. Observed charge envelope and average DAR value (calculated from triplicate injection on RPLC-MS) for vcMMAE-Trastuzumab for samples subjected to reduction for 30 minutes, 60 minutes, 2 hours and 4 hours.


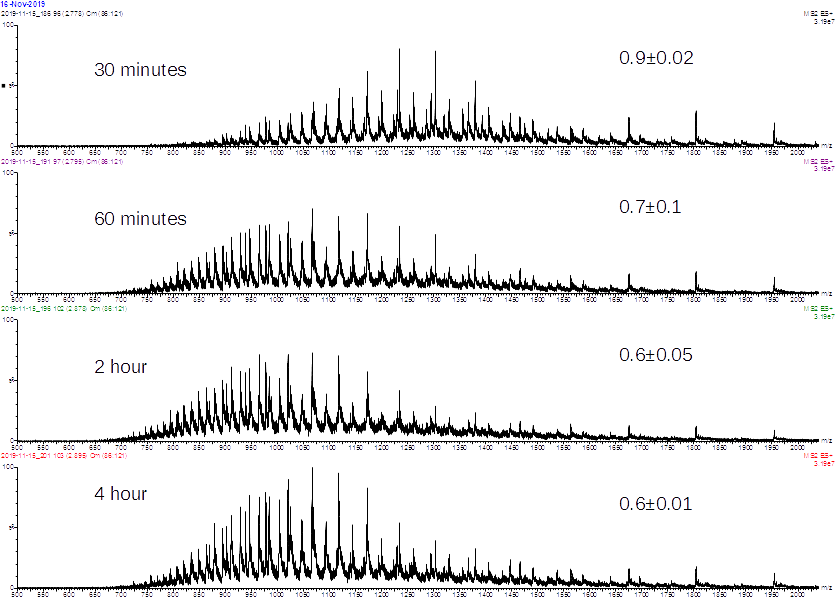


Figure S - 4. Observed charge envelope and average DAR value (calculated from triplicate injection on RPLC-MS) for DM1-Trastuzumab for samples subjected to reduction for 30 minutes, 60 minutes, 2 hours and 4 hours.

## Desalting (Ionization)


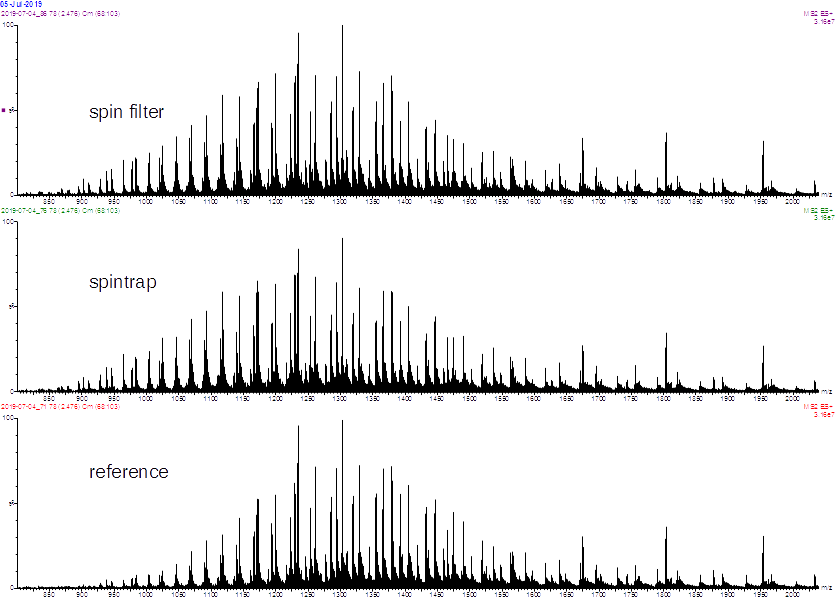


Figure S - 5. Mass spectrum of DM1-Trastuzumab ADC sample from desalting by either spin traps or spin filters or none of the above.


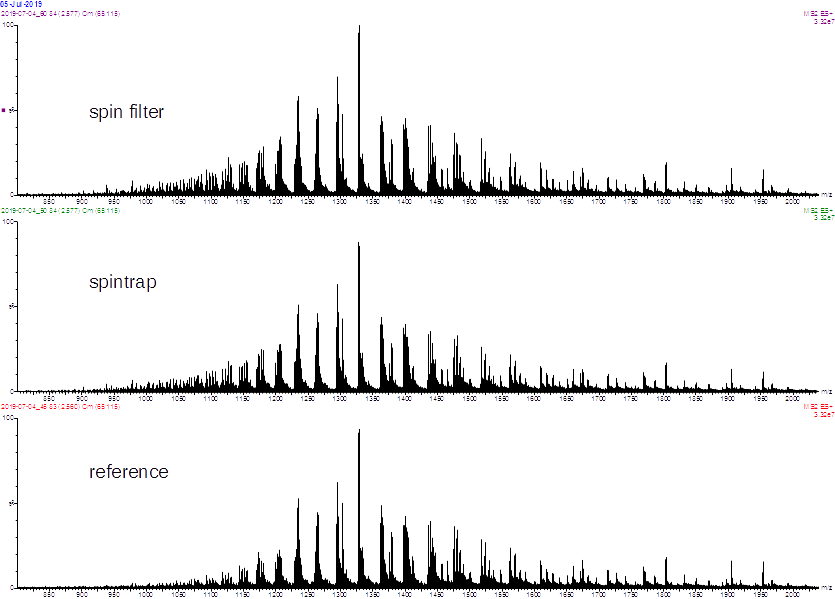


Figure S - 6. Mass spectrum of vcMMAE-Trastuzumab ADC sample from desalting by either spin traps or spin filters or none of the above.


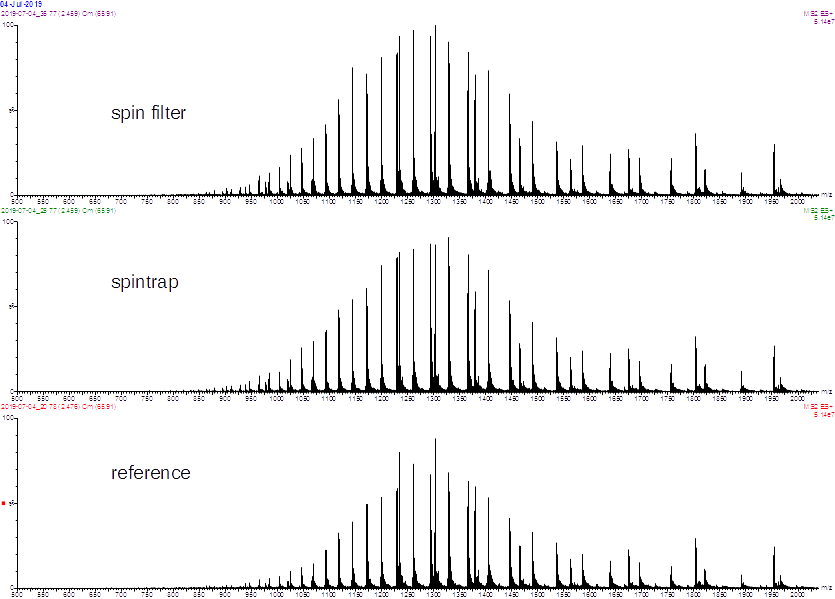


Figure S - 7. Mass spectrum of Trastuzumab desalted by spin-filter (top) and spintrap (middle) or non-treated (bottom).

## Deconvoluted mass spectra (ADCs)


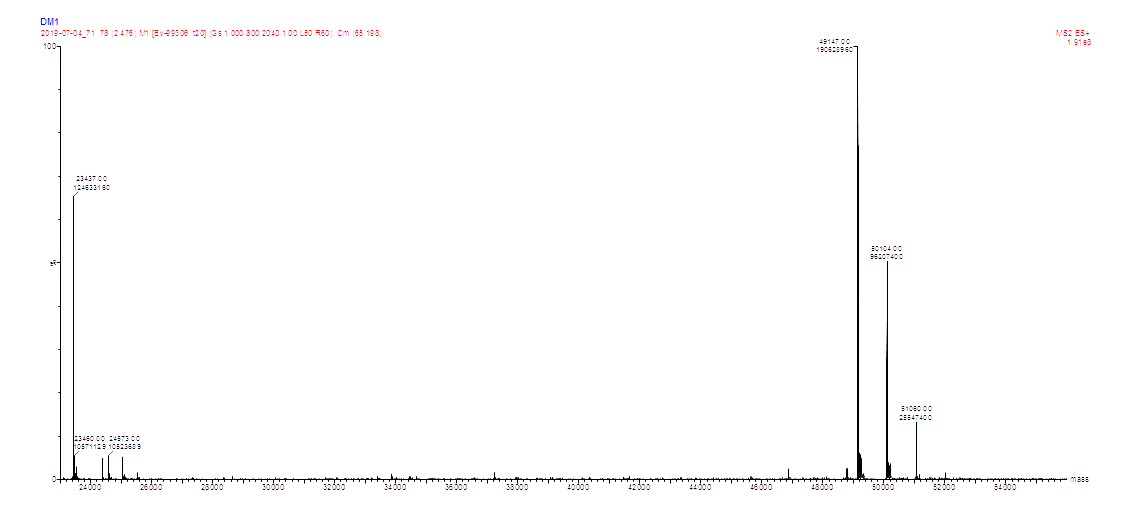


Figure S - 8. Deconvoluted spectrum over light and heavy chains of deglycosylated and reduced DM1-Trastuzumab.


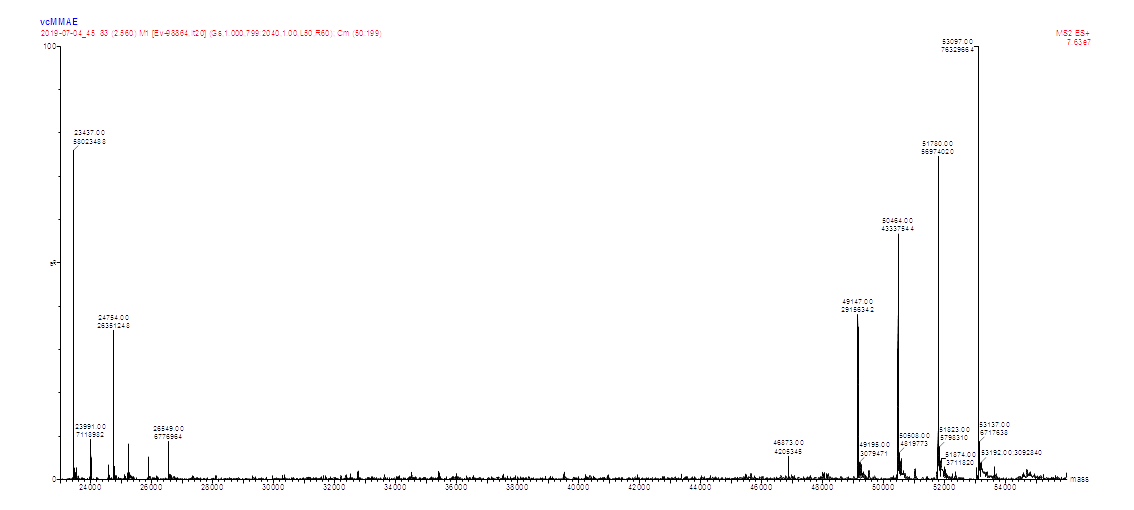


Figure S - 9. Deconvoluted spectrum over light and heavy chains of deglycosylated and reduced vcMMAE-Trastuzumab.

## Desalting (recovery)

Table S - 1. Recovery of signal (compared to sample without desalting step) from samples desalted prior to the reduction step, for injecting the corresponding amount of sample into the RPLC-MS. Values are calculated taking the dilution of desalted samples for duplicate samples preparations.

| Device | **Trastuzumab** | | | **DM1** | | | **vcMMAE** | | |
| --- | --- | --- | --- | --- | --- | --- | --- | --- | --- |
|  | **Recovery** | **±** | **p-value** | **Recovery** | **±** | **p-value** | **Recovery** | **±** | **p-value** |
| Spin filter | 120% | 6% | < 0.05 | 113% | 5% | < 0.05 | 128% | 1% | < 0.05 |
| Spintrap | 128% | 12% | < 0.05 | 99% | 1% | 0.53 | 114% | 1% | < 0.05 |

p-values are from one-way ANOVA compared to non-desalted

± column state the standard deviations for recovery of both preparative and analytical replicates

Table S - 2. Recovery of signal (compared to sample without desalting step) from samples desalted after the reduction step, for injecting the corresponding amount of sample into the RPLC-MS. Values are calculated taking the dilution of desalted samples for duplicate samples preparations, except for Trastuzumab.

| Device | **Trastuzumab** | | | **DM1** | | | **vcMMAE** | | |
| --- | --- | --- | --- | --- | --- | --- | --- | --- | --- |
|  | **Recovery** | **±** | **p-value** | **Recovery** | **±** | **p-value** | **Recovery** | **±** | **p-value** |
| Spin filter | 85% | 1% | 0.15 | 139% | 7% | < 0.05 | 105% | 8% | 0.48 |
| Spintrap | 93% | 1% | 0.16 | 102% | 3% | 0.16 | 81% | 7% | < 0.05 |

p-values are from one-way ANOVA compared to non-desalted
± column state the standard deviations for recovery of both preparative and analytical replicates

Table S - 3. Recovery calculated from Bradford data for samples desalted before and after the reduction step.

|  | **Trastuzumab** | | | | **DM1-Trastuzumab** | | | | **vcMMAE-Trastuzumab** | | | |
| --- | --- | --- | --- | --- | --- | --- | --- | --- | --- | --- | --- | --- |
|  | **Before** | **±** | **After** | **±** | **Before** | **±** | **After** | **±** | **Before** | **±** | **After** | **±** |
| **Spin-filter** | 95% | 4% | N/A | | 96% | 0% | 97% | 11% | 99% | 5% | 106% | 1% |
| **Spintrap** | 84% | 2% | N/A | | 78% | 7% | 93% | 2% | 76% | 3% | 87% | 1% |

## Storage

Table S - 4. Percentile change in how large percentage of the total signal constituted of aggregate, monomer and fragment signal for Trastuzumab after 7 or 41-44 days storage at two different temperatures.

|  | **+4°C** | | **-20°C** | |
| --- | --- | --- | --- | --- |
|  | **7 days storage** | **41 days storage** | **7 days storage** | **44 days storage** |
| Aggregates | 0.3% | -0.2% | 0.9% | 0.2% |
| Monomer | -0.6% | -5.1% | -1.2% | -0.2% |
| Fragments | 0.3% | 5.2% | 0.3% | -0.03% |

Table S - 5. Percentile change in how large percentage of the total signal constituted of aggregate, monomer and fragment signal for DM1-Trastuzumab ADC after 7 or 41-44 days storage at two different temperatures.

|  | **+4°C** | | **-20°C** | |
| --- | --- | --- | --- | --- |
|  | **7 days storage** | **41 days storage** | **7 days storage** | **44 days storage** |
| Aggregates | 0.5% | 0.8% | 1.5% | 0.7% |
| Monomer | -0.8% | -1.6% | -1.8% | -0.7% |
| Fragments | 0.3% | 0.8% | 0.3% | 0.02% |

Table S - 6. Percentile change in how large percentage of the total signal constituted of aggregate, monomer and fragment signal for vcMMAE-Trastuzumab ADC after 7 or 41-44 days storage at two different temperatures.

|  | **+4°C** | | **20°C** | |
| --- | --- | --- | --- | --- |
|  | **7 days storage** | **41 days storage** | **7 days storage** | **44 days storage** |
| Aggregates | -0.8% | -0.8% | -0.1% | -0.8% |
| Monomer | 0.9% | -12.1% | 0.1% | 7.0% |
| Fragments | -0.1% | 12.9% | 0.04% | -6.25% |
